# Extent of Endobronchial Ultrasound Nodal Sampling and Risk of Pathologic N2 Upstaging in Clinical N0–N1 Non‐Small Cell Lung Cancer

**DOI:** 10.1111/1759-7714.70365

**Published:** 2026-09-10

**Authors:** Min Jee Kim, Jae Cheol Lee, Chang‐Min Choi, Sei Won Lee, Jae Seung Lee, Kyung‐Wook Jo, Ho Cheol Kim, Jee Hwan Ahn, Jang Ho Lee, Dong‐Gon Hyun, Wonjun Ji

**Affiliations:** ^1^ Department of Pulmonary and Critical Care Medicine Asan Medical Center, University of Ulsan College of Medicine Seoul South Korea; ^2^ Department of Oncology Asan Medical Center, University of Ulsan College of Medicine Seoul South Korea

**Keywords:** sampling extent, staging endobronchial ultrasound, upstaging N2

## Abstract

**Background:**

Endobronchial ultrasound (EBUS)–guided transbronchial needle aspiration (TBNA) is strongly recommended for mediastinal staging in resectable non–small cell lung cancer (NSCLC). However, the optimal sampling extent remains uncertain. This study aimed to evaluate whether the extent of nodal sampling during staging EBUS is associated with pathologic N2 upstaging in clinical N0–N1 NSCLC.

**Methods:**

This retrospective cohort included patients with cN0–N1 NSCLC who underwent preoperative EBUS‐TBNA between January 2022 and December 2023. Patients were stratified into three groups according to the number of sampled lymph node stations at the N2/N3 levels: Group 1, ≤ 1 mediastinal station at N2 or N3; Group 2, one N2 and one N3 station; Group 3, ≥ 3 stations, including at least one N2 and one N3. The primary outcome was pathologic N2 upstaging, defined as N2 disease confirmed on surgical pathology despite preoperative stage N0–N1.

**Results:**

A total of 418 patients were included (mean age, 68.6 years; 64.1% male). Most had adenocarcinoma (75.4%) and cN0 disease (94.7%). Group distribution was 140 (33.5%) in Group 1, 92 (22.0%) in Group 2, and 186 (44.5%) in Group 3. Pathologic N2 upstaging occurred in 21/140 (15.0%) in Group 1, 8/92 (8.7%) in Group 2, and 8/186 (4.3%) in Group 3. The rate decreased with increasing sampling, with Group 3 showing a lower rate than Group 1 (*p* = 0.005).

**Conclusions:**

Comprehensive staging EBUS‐TBNA with ≥ 3 N2/N3 stations was associated with a lower rate of occult N2 upstaging in clinical N0–N1 NSCLC, underscoring the importance of comprehensive nodal evaluation for accurate staging.

## Graphical abstract

Greater extent of EBUS nodal sampling was associated with progressively lower rates of unexpected pathologic N2 disease. Sampling of ≥ 3 mediastinal stations at least one N2 and N3 showed the lowest upstaging risk compared with limited sampling.

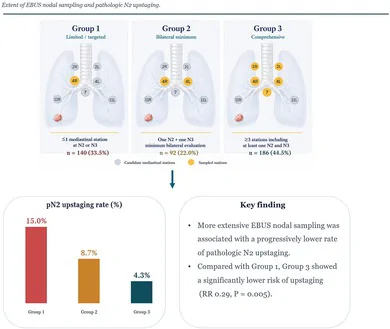

## 1 Introduction

The introduction of low‐dose computed tomography (LDCT) for lung cancer screening has led to a substantial reduction in lung cancer–specific mortality and improved overall survival among patients with non–small cell lung cancer (NSCLC) [1, 2]. This benefit is largely attributable to increased detection of surgically resectable early‐stage disease, resulting in a stage shift toward earlier diagnoses [3, 4]. Furthermore, neoadjuvant chemoimmunotherapy has markedly improved survival outcomes in patients with node‐positive stage II–III NSCLC who are at high risk of recurrence following surgery [5, 6]. Therefore, accurate preoperative staging is essential for optimal treatment planning and prognosis in patients with surgically resectable NSCLC.

Although computed tomography (CT) and positron emission tomography (PET‐CT) provide valuable preoperative information, their sensitivity and specificity for mediastinal nodal staging are limited. Several studies have reported that a substantial proportion of patients with radiologically normal mediastinum harbor occult N2 disease, particularly those with central tumors, cT2 lesions (≥ 3 cm), or clinical N1 involvement [7, 8]. Consequently, endobronchial ultrasound‐guided transbronchial needle aspiration (EBUS‐TBNA) has become an indispensable, minimally invasive modality for mediastinal staging in resectable NSCLC [9]. Recent international guidelines recommend systematic EBUS‐TBNA and/or endoscopic ultrasound (EUS) in patients at high risk for mediastinal metastasis, even when mediastinal lymph nodes appear normal on imaging [10].

Although recent large‐scale studies have suggested that comprehensive multi‐station endosonographic staging may improve diagnostic yield over targeted approaches [11, 12], the optimal number of nodal sampling and the extent of sampling remain unclear [7, 8, 13, 14, 15, 16]. In particular, the appropriate extent of N2/3 nodal evaluation is not well established in patients with clinical N0–N1 NSCLC without radiologic evidence of N2 involvement.

Therefore, this study aimed to evaluate the association between the extent and number of N2/3 stations sampled during EBUS‐guided nodal staging and the rate of pathologic N2 upstaging following surgery in patients with resectable NSCLC, and to explore the optimal extent of mediastinal nodal staging.

## 2 Methods

### 2.1 Study Design and Population

This single‐center retrospective study was conducted at Asan Medical Center, a tertiary hospital in South Korea, between January 1, 2022, and December 31, 2023, to evaluate the impact of the extent of nodal staging on the rate of unforeseen N2 disease in patients with NSCLC. Eligible patients were those who underwent surgical resection following preoperative mediastinal lymph node staging with EBUS‐TBNA. Patients were excluded if they had received neoadjuvant chemotherapy, radiotherapy, or immunotherapy, had incomplete pathological staging after resection, had a prior history of lung cancer or other thoracic malignancies, or had incomplete clinical records.

EBUS‐TBNA was performed when mediastinal staging was clinically indicated, in accordance with current guidelines [10]. Specifically, indications included: [1] enlarged mediastinal or hilar lymph nodes (short‐axis diameter ≥ 10 mm) on chest CT or PET‐CT; [2] increased fluorodeoxyglucose (FDG) uptake in mediastinal or hilar lymph nodes on PET‐CT (PET avid); [3] central tumor location, tumor ≥ 3 cm, or clinical N1 disease, even in the absence of suspicious mediastinal findings on imaging; and [4] other cases at the discretion of the multidisciplinary tumor board where mediastinal evaluation was deemed necessary before surgery. The study was approved by the Asan Medical Center Institutional Review Board (IRB No.: 2024–0238). Informed consent requirements were waived due to the retrospective nature of the study.

### 2.2 Data Collection

Demographic and clinical data were extracted from electronic medical records, including age, sex, body mass index (BMI), smoking history, and the maximum standardized uptake value (SUVmax) of the primary tumor on PET‐CT. Tumor‐related variables, including histological subtype, tumor location, and clinical stage, were also recorded. A central tumor was defined as one with its epicenter located within the inner one‐third of the hemithorax on axial CT images, delineated by concentric lines from the midline toward the lateral pleural surface. Lesions with epicenters beyond this zone were classified as peripheral [17]. Procedural variables included the number and location of mediastinal lymph node stations sampled during EBUS‐TBNA and the results of cytopathologic analysis. EBUS‐TBNA was performed according to routine clinical practice, with target lymph node stations selected based on preoperative imaging findings and the operator's clinical judgment and procedural feasibility. The institutional protocol aimed to obtain at least two adequate core tissue samples from each targeted lymph node. When necessary, three to four EBUS‐TBNA passes were performed per station. Rapid on‐site evaluation (ROSE) was not routinely used at our center. The extent of surgical resection and the final pathologic stage were also reviewed. Clinical and final pathologic stages were assigned based on the 8th edition of the TNM classification.

Among patients with preoperative clinical N0–1 disease, those who underwent staging EBUS were categorized into three groups (Groups 1–3) according to the number of sampled lymph node stations at the N2 or higher level: Group 1, ≤ 1 mediastinal station at N2 or N3; Group 2, one N2 and one N3 station; and Group 3, ≥ 3 stations, including at least one N2 and one N3 station. The categorization based on the number of sampled nodal stations was designed to reflect the extent of mediastinal sampling in real‐world clinical practice, rather than to assess adherence to guideline‐defined systematic mediastinal staging. Although sampling of ≥ 3 stations, including at least one N2 and one N3 station, may partially align with the general principles of systematic mediastinal staging, this approach was intended to reflect sampling extent rather than formal adherence to guideline‐recommended systematic EBUS [16].

Patients with suspected metastasis to EBUS‐inaccessible lymph node stations (e.g., stations 5 and 6) on preoperative evaluation were excluded. Suspicion was based on preoperative chest CT and PET‐CT findings, including lymph node enlargement (short‐axis diameter ≥ 10 mm on CT) or PET‐CT findings considered suspicious for nodal metastasis. PET‐CT findings were interpreted in the clinical context, as a uniform SUV cutoff could not be applied. Nodal FDG uptake was assessed in relation to the metabolic activity of the primary tumor, particularly in cases with low primary tumor uptake, based on interpretation by nuclear medicine physicians. These imaging findings were subsequently reviewed in multidisciplinary team discussions.

The primary outcome was unforeseen N2, defined as pathological N2 disease identified at surgical resection in patients preoperatively staged as cN0–1 by imaging and EBUS‐TBNA. This definition also included cases in which EBUS‐inaccessible lymph node stations (e.g., stations 5 and 6) were assessed as negative on preoperative evaluation but were unexpectedly found to be positive after surgery, reflecting routine clinical staging practice.

### 2.3 Statistical Analysis

Categorical variables were summarized as frequencies and percentages, and continuous variables as means with standard deviations or medians with interquartile ranges, depending on distribution. Between‐group comparisons were performed using chi‐square (χ^2^) or Fisher's exact tests for categorical variables and ANOVA or Kruskal–Wallis tests for continuous variables.

To evaluate the association between the extent of EBUS‐TBNA sampling (1, 2, or ≥ 3 stations) and the risk of unforeseen N2 upstaging, overall differences among the three groups were examined using the χ^2^ test for independence. When a significant overall difference was observed, pairwise comparisons of proportions were performed, with resulting *p*‐values adjusted for multiple testing using the Bonferroni correction. Trends across ordered categories were assessed by modeling sampling extent as an ordinal predictor in logistic regression, reporting the Wald *p* value as the *p* for trend. The trend analysis was performed both without adjustment and with adjustment for SUVmax and clinical N stage. Multivariable logistic regression analysis was conducted to identify independent predictors of unforeseen N2 upstaging, adjusting for relevant demographic and clinical covariates. All tests were two‐sided, and *p* < 0.05 was considered statistically significant. Analyses were performed using R version 4.2.1 (R Foundation for Statistical Computing, Vienna, Austria).

## 3 Results

### 3.1 Baseline Characteristics

Table 1 summarizes the baseline characteristics of the study population. A total of 418 patients with clinical N0–1 NSCLC were included, comprising 140 in Group 1, 92 in Group 2, and 186 in Group 3. The mean age of the cohort was 68.6 ± 7.9 years, and 64.1% of patients were male. Regarding smoking history, 43.5% were never smokers, 34.2% were former smokers, and 22.2% were current smokers, with a mean of 32.8 ± 23.6 pack‐years among ever smokers (*p* = 0.098).

**TABLE 1:** Baseline characteristics according to the extent of EBUS nodal sampling in patients with clinical N0–1 NSCLC.

Characteristic	Total	Group 1	Group 2	Group 3	*p*
Number of patients	418	140	92	186	
Age	68.6 ± 7.9	68.1 ± 8.8	69.1 ± 8.0	68.7 ± 7.2	0.660
Male	268 (64.1)	81 (57.9)	66 (71.7)	121 (65.1)	0.092
BMI	23.7 ± 3.0	23.7 ± 3.0	23.8 ± 3.3	23.6 ± 2.8	0.885
Smoking history					0.117
Never smoker	182 (43.5)	71 (50.7)	34 (37.0)	77 (41.4)	
Former smoker	143 (34.2)	39 (27.9)	40 (43.5)	64 (34.4)	
Current smoker	93 (22.2)	30 (21.4)	18 (19.6)	45 (24.2)	
Pack‐years	32.8 ± 23.6	29.7 ± 17.8	29.9 ± 17.9	36.4 ± 28.7	0.098
Histology					0.609
Adenocarcinoma	315 (75.4)	110 (78.6)	69 (75.0)	136 (73.1)	
Squamous cell carcinoma	90 (21.5)	28 (20.0)	19 (20.7)	43 (23.1)	
Others	13 (3.1)	2 (1.4)	4 (4.3)	7 (3.8)	
Tumor size > 3 cm	216 (51.7)	78 (55.7)	47 (51.1)	91 (48.9)	0.475
SUVmax of the primary tumor, PET‐CT	7.8 ± 5.5	7.9 ± 5.4	8.0 ± 5.6	7.6 ± 5.7	0.845
Central location*	127 (30.4)	45 (32.1)	25 (27.2)	57 (30.6)	0.719
Clinical N stage					0.179
cN0	396 (94.7)	129 (92.1)	87 (94.6)	180 (96.8)	
cN1	22 (5.3)	11 (7.9)	5 (5.4)	6 (3.2)	

*Note:* Data are presented as mean ± standard deviation, or number (%).

Abbreviations: BMI, body mass index; EBUS, endobronchial ultrasound; NSCLC, non–small cell lung cancer; PET‐CT, positron emission tomography–computed tomography; SUVmax, maximum standardized uptake value.

* Central tumor location was defined as involvement of the inner one‐third of the hemithorax on axial CT scans.

Adenocarcinoma was the most common histological subtype (75.4%), followed by squamous cell carcinoma (21.5%). Tumors > 3 cm were observed in 51.7% of patients, and 30.4% had centrally located tumors. The mean SUVmax on PET‐CT was 7.8 ± 5.5. Most patients were staged as cN0 (94.7%) at presentation, while 5.3% were staged as cN1.

Most baseline clinical characteristics, including tumor size, SUVmax, and tumor location, did not differ significantly among the three groups. The distribution of clinical N stage also did not differ significantly across the three groups (*p* = 0.179) (Table 1).

### 3.2 Rate of Unforeseen Pathologic N2

Among the 418 patients, pathologic N2 upstaging occurred in 37 cases (8.9%). The upstaging rate was significantly higher in patients with sampling limited to one or fewer N2/N3 stations compared with patients in whom more than two stations were sampled (21/140 vs. 16/278, *p* = 0.003). Stratified by sampling extent, the upstaging rate decreased with greater sampling extent: 15.0% in Group 1 (21/140), 8.7% in Group 2 (8/92), and 4.3% in Group 3 (8/186), showing a significant linear trend (odds ratio per increasing sampling category, 0.51; 95% CI, 0.33–0.76; *p* for trend = 0.001). The trend remained significant after adjustment for SUVmax and clinical N stage (adjusted odds ratio per increasing sampling category, 0.56; 95% CI, 0.36–0.86; *p* for trend = 0.010).

**FIGURE 1:**
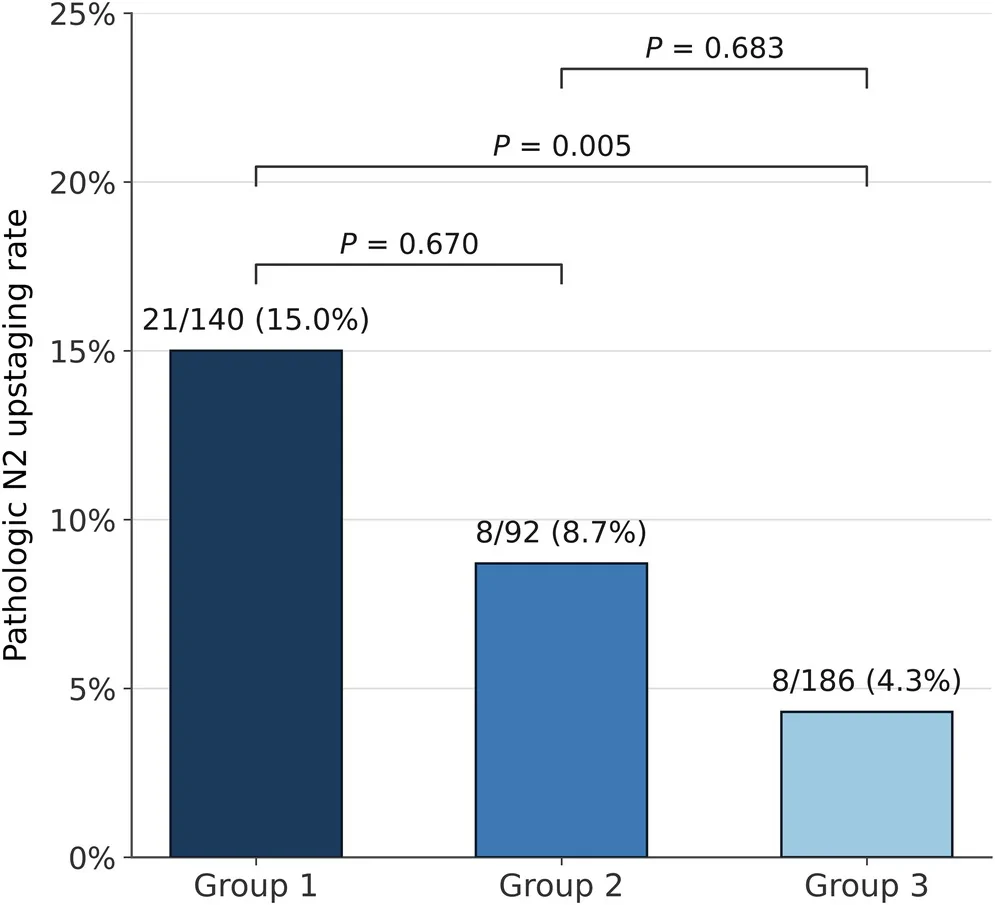
Rate of pathologic N2 upstaging by extent of EBUS nodal sampling.

In pairwise comparisons with Group 1, Group 2 showed a lower but not statistically significant upstaging rate, with an absolute risk reduction of 6.3 percentage points (95% CI, −2.0 to 14.6) and a relative risk of 0.58 (95% CI, 0.27–1.25). Group 3 demonstrated a significantly lower upstaging rate than Group 1 (*p* = 0.005; Bonferroni‐adjusted), with a relative risk of 0.29 (95% CI, 0.13–0.63) and an absolute risk reduction of 10.7 percentage points (95% CI, 4.1–17.3) (Table 2) (Figure 1).

**TABLE 2:** Pathologic upstaging N2 rate according to group stratification by the extent of EBUS nodal sampling in patients with clinical N0‐1 NSCLC.

Extent	Number of patients	Pathologic N2 upstaging	Upstaging rate	Relative risk (95% CI)	Absolute risk reduction (95% CI)	Pairwise *p* *	*p* for trend
Overall	418	37	8.9%	—	—		0.001
Group 1	140	21	15.0%	Ref	Ref	Ref	
Group 2	92	8	8.7%	0.58 (0.27–1.25)	6.3 (−2.0–14.6)	0.670	
Group 3	186	8	4.3%	0.29 (0.13–0.63)	10.7 (4.1–17.3)	0.005	

Abbreviations: CI, confidence interval; EBUS, endobronchial ultrasound; NSCLC, non–small cell lung cancer.

* Pairwise *p* value were Bonferroni‐adjusted. *p* for trend was derived from a univariable logistic regression model with sampling extent modeled as an ordinal predictor (Group 1 = 1, Group 2 = 2, Group 3 = 3), using the Wald test.

### 3.3 Risk Factors for Unforeseen Pathologic N2

In the unadjusted logistic analysis, higher SUVmax of primary tumor and clinical N1 status were significantly associated with a higher risk of pathologic N2 upstaging (SUVmax: OR, 1.077; 95% confidence interval [CI], 1.019–1.138; *p* = 0.007; cN1 vs. cN0: OR, 4.415; 95% CI, 1.497–11.610; *p* = 0.004), whereas more extensive sampling was associated with a lower risk (Group 3 vs. Group 1: OR, 0.255; 95% CI, 0.103–0.573; *p* = 0.002) (Table 3).

**TABLE 3:** Risk factors for unforeseen pathologic N2 upstaging in patients with clinical N0–1 NSCLC.

Characteristics	Unadjusted	*p*	Multivariable	*p*
	OR (95% CI)		OR (95% CI)	
Age	0.950 (0.913–0.988)	0.009		
Tumor size > 3 cm	1.414 (0.717–2.859)	0.323		
SUVmax of the primary tumor	1.077 (1.019–1.138)	0.007	1.073 (1.013–1.135)	0.014
Tumor location				
Peripheral	Reference	—		
Central	0.836 (0.374–1.731)	0.642		
Clinical N stage				
cN0	Reference	—	Reference	—
cN1	4.415 (1.497–11.610)	0.004	2.679 (0.788–7.792)	0.086
Group				
1	Reference	—	Reference	—
2	0.540 (0.216–1.234)	0.160	0.591 (0.218–1.451)	0.270
3	0.255 (0.103–0.573)	0.002	0.313 (0.122–0.737)	0.010

*Note:* Odds ratios were estimated using univariable and multivariable logistic regression; 95% confidence intervals are profile‐likelihood intervals. The multivariable model included SUVmax, clinical N stage, and sampling group, and was fitted in 375 patients with complete SUVmax data.

Abbreviations: cN, clinical N stage; NSCLC, non–small cell lung cancer; OR, odds ratio; SUVmax, maximum standardized uptake value.

In the multivariable analysis, higher SUVmax of the primary tumor remained independently associated with a higher risk of pathologic N2 upstaging (adjusted OR [aOR], 1.073; 95% CI, 1.013–1.135; *p* = 0.014). Clinical N1 disease showed a numerically higher odds of upstaging in the multivariable analysis, although the association did not reach statistical significance (cN1 vs. cN0: aOR, 2.679; 95% CI, 0.788–7.792; *p* = 0.086).

Regarding sampling extent, Group 3 remained significantly associated with a reduced risk of upstaging compared with Group 1 (aOR, 0.313; 95% CI, 0.122–0.737; *p* = 0.010), whereas Group 2 did not differ significantly from Group 1 (aOR, 0.591; 95% CI, 0.218–1.451; *p* = 0.270). The direction of the association between sampling extent and upstaging remained consistent with the univariable results and the observed upstaging rates.

## 4 Discussion

This study demonstrates that in clinical N0–1 NSCLC, more comprehensive nodal sampling during staging EBUS‐TBNA was associated with a lower risk of unforeseen pathologic N2 disease. The upstaging rate declined progressively with increasing sampling extent, from 15.0% with ≤ 1 station to 4.3% with ≥ 3 stations, highlighting the importance of comprehensive nodal evaluation. Additionally, higher SUVmax was identified as an independent predictor of occult N2 metastasis, whereas more extensive sampling of ≥ 3 mediastinal stations was associated with a significantly lower risk.

Accurate mediastinal staging is essential for establishing appropriate treatment strategies in NSCLC. Current international guidelines recommend minimally invasive endosonographic techniques, such as EBUS‐TBNA and EUS‐guided fine‐needle aspiration, as the preferred approaches. Although systematic nodal staging is not strictly defined, it generally involves sequential evaluation from N3 to N1 stations. There is a broad consensus that both N2 and N3 nodes should be examined whenever feasible; however, the extent of sampling and selection of target stations remains controversial and has yet to be standardized [13, 16].

Despite guideline recommendations, adherence to systematic EBUS‐TBNA remains inconsistent in real‐world practice. Some studies suggest that targeted sampling may be non‐inferior to systematic EBUS [15, 18]. International surveys indicate that only approximately two‐thirds of interventional bronchoscopists perform systematic evaluation [19], and fewer than half of general pulmonologists were able to perform adequate systematic mediastinal assessment in a proctored simulated EBUS staging study [20]. Furthermore, a meta‐analysis of patients with radiologically normal mediastinum demonstrated that nearly half underwent sampling of fewer than two lymph nodes [21]. This discrepancy between guideline recommendations and real‐world practice underscores a critical gap in mediastinal staging. Incomplete or selective nodal assessment may compromise staging accuracy and increase the risk of undetected N2 disease. Therefore, additional evidence is required to reinforce the value of comprehensive staging and to clarify the optimal extent and number of stations for reliable clinical application.

Sampling of three or more mediastinal stations, including both N2 and N3, was associated with a significantly lower rate of occult N2 upstaging in patients with clinical N0–1 NSCLC, supporting the diagnostic advantage of comprehensive mediastinal evaluation. Several previous studies, including the large prospective SCORE study [13] and more recent multicenter analyses [11, 12], have consistently demonstrated that systematic EBUS provides superior staging accuracy compared with targeted approaches, emphasizing the importance of a systematic nodal assessment. In this context, mediastinal staging should extend beyond radiographically suspicious nodes to include contralateral N3 assessment whenever feasible. In a retrospective cohort of patients with cN2–N3 disease, systematic EBUS modified nodal classification in 13% of cases and identified occult N3 metastases in 3.8%, findings that would directly influence surgical eligibility [15]. Furthermore, surgical series have shown that the extent of nodal evaluation—measured by the number of nodes or stations examined—is independently associated with improved staging accuracy and long‐term survival [22, 23]. Collectively, these findings support the clinical relevance of distinguishing limited from more extensive multi‐station sampling.

In our study, patients in whom only one of the N2 or N3 stations was sampled, regardless of the total number of stations, had a higher rate of pN2 upstaging than those in whom both N2 and N3 stations were included. The upstaging rate declined progressively with sampling of two and then three or more N2/N3 stations. Although the two‐station group did not differ significantly from the single‐station group, the overall trend remained significant. The lowest upstaging rate was observed in patients who underwent sampling of three or more stations, including both N2 and N3 stations, supporting the importance of broader mediastinal coverage. Cases with only one or fewer N2 or N3 stations sampled likely reflected limited radiographically suspicious nodes or technical constraints. However, such limited sampling inherently increases the likelihood of incomplete staging. Notably, even in the presence of radiographically negative mediastinal nodes, occult N2 disease may still be present [15, 24, 25]. These findings emphasize that comprehensive sampling, regardless of radiographic findings, is critical to minimize the risk of understaging and to provide more reliable staging outcomes for clinical decision‐making. Sampling of three or more stations, including at least one N2 and one N3, reflects a more extensive mediastinal evaluation [10] and improves the reliability of nodal assessment. In addition, negative findings at N3 stations suggest a lower likelihood of occult N2 disease. For example, Lucena et al. reported that the risk of occult N2/N3 disease after negative EBUS and mediastinoscopy was approximately 4.4%, indicating that thorough assessment of mediastinal lymph nodes, including N3 stations, can substantially reduce missed metastases [8]. Similarly, Al‐Sarraf et al. observed that among patients clinically staged as negative for N2/N3 disease by PET‐CT, approximately 16% nonetheless harbored occult mediastinal lymph node metastasis, highlighting the importance of negative verification at N3 in reducing occult N2 disease [24]. Therefore, incorporating N3 nodes into comprehensive staging may enhance staging completeness and reduce the risk of missed metastases.

However, these findings should be interpreted with caution because the extent of EBUS sampling was not randomly assigned but was determined by clinical judgment. In particular, Group 3 had a lower proportion of clinical N1 disease than Groups 1 and 2. Although clinical N1 status did not reach statistical significance after adjustment, it showed a numerically higher odds of pN2 upstaging and therefore may have contributed, at least in part, to the observed difference across groups. Nevertheless, sampling of three or more stations, including both N2 and N3 stations, remained significantly associated with a lower risk of pN2 upstaging in the multivariable analysis. These findings suggest that the lower upstaging rate observed in Group 3 may not be fully explained by baseline clinical N‐stage imbalance alone.

Taken together, our findings suggest that more extensive nodal sampling may improve staging completeness and reduce the likelihood of missed occult N2 disease. Nevertheless, given the observational design and nonrandomized sampling extent, the observed relationship should be interpreted as an association rather than a causal effect. The consistency of this association after adjustment supports the potential clinical value of comprehensive nodal evaluation, particularly when both N2 and N3 stations are included.

In contrast to the previous study that identified central tumor location as a significant predictor of nodal metastasis [26], central tumor location was not significantly associated with pathologic N2 upstaging in our univariable analysis (OR, 0.836; 95% CI, 0.374–1.731; *p* = 0.642). Shin et al., in a study including 1337 patients with radiologic N0 disease, suggested defining central tumors as those with epicenters located within the inner one‐third of the hemithorax, determined by drawing concentric lines from the midline [17]. However, a subsequent study by Choi et al., involving 436 patients with resectable adenocarcinoma, concluded that this definition may not be universally applicable or reliable for predicting nodal involvement [27]. The lack of consistency in defining central location across studies has contributed to ongoing debate regarding its prognostic significance. Similarly, in the Korean prospective multicenter PLUS study [28], which developed prediction models for mediastinal metastasis and detection by EBUS‐TBNA in potentially resectable NSCLC, central tumor location was included as a candidate variable but contributed minimally to overall risk estimation. Moreover, in the model predicting nodal metastasis detection by EBUS‐TBNA, central tumor location was not statistically significant in multivariable analysis. These findings suggest that the prognostic impact of central tumor location remains controversial. Further large, prospective studies are warranted to elucidate its prognostic and clinical implications.

This study has some limitations. First, it was conducted at a single center with a relatively small sample size, which may limit the generalizability and reduce statistical power. Second, the sampling categories were based on the number and distribution of sampled N2/N3 stations and therefore represent study‐specific measures of sampling extent rather than formal adherence to guideline‐defined systematic mediastinal staging. Third, as a nonrandomized observational study, the extent of EBUS‐TBNA sampling was not standardized and was determined at the operator's discretion rather than by a predefined protocol. Accordingly, patients who underwent more extensive sampling may have differed from those who underwent limited sampling in baseline clinical characteristics. The decision to sample additional stations may have been influenced by the operator's clinical assessment of nodal risk, subtle radiologic findings, patient condition, or technical feasibility, thereby introducing potential selection bias, confounding by indication, and operator‐dependent variability. Consequently, the observed association between sampling extent and nodal upstaging should be interpreted with caution and cannot be considered causal. Fourth, although the number of sampled stations reflects the extent of nodal evaluation, this metric does not capture qualitative procedural factors, such as the number of needle passes, needle gauge, adequacy of tissue acquisition, or the use of ROSE. These unmeasured procedural factors may have influenced diagnostic performance. In addition, because lymph node stations 5 and 6 are not accessible by EBUS‐TBNA, patients with suspected metastasis to these stations were excluded. Although postoperative detection of metastasis in these stations despite negative preoperative assessment was classified as unforeseen N2 disease to reflect real‐world staging outcomes, such cases are not directly attributable to the extent of EBUS sampling and may have influenced the observed association. Fifth, the present analysis was limited to EBUS‐TBNA and did not include complementary endosonographic modalities, such as esophageal ultrasound performed using the EBUS scope (EUS‐B). The prospective multicenter SCORE trial demonstrated that systematic combined endosonography (EBUS plus EUS‐B) significantly enhanced the sensitivity of mediastinal staging compared with targeted endosonography [13]. Therefore, the addition of EUS‐B may further improve staging completeness, particularly for stations 8 and 9, and warrants consideration in future studies of comprehensive mediastinal evaluation. Finally, verification bias is possible because not all mediastinal nodal stations underwent uniform pathological confirmation, raising the possibility that some negative findings represent missed disease.

In conclusion, our findings indicate that sampling at least three N2/N3 stations during preoperative staging EBUS‐TBNA is associated with a lower risk of occult pathologic N2 upstaging in patients with clinical N0–N1 NSCLC. Comprehensive nodal assessment further enhances staging completeness and reinforces the reliability of negative results.

## Author Contributions

Study concept and design: M.J. Kim and W. Ji. Data collection: M.J. Kim, J.C. Lee, C.‐M. Choi, S.W. Lee, J.S. Lee, K.W. Jo, H.C. Kim, J.H. Lee, J.H. Ahn, D.G. Hyun, and W. Ji. Data analysis and interpretation: M.J. Kim and W. Ji. Drafting of the manuscript: M.J. Kim and W. Ji. Critical revision of the manuscript: M.J. Kim, J.C. Lee, C.‐M. Choi, S.W. Lee, J.S. Lee, K.W. Jo, H.C. Kim, J.H. Lee, J.H. Ahn, D.G. Hyun, and W. Ji. All authors have read and approved the final manuscript.

## Funding

This study was supported by a grant (No. 2024IL0014) from the Asan Institute for Life Sciences, Asan Medical Center, Seoul, Korea.

## Conflicts of Interest

The authors declare no conflicts of interest.

## Data Availability

The data that support the findings of this study are available from the corresponding author upon reasonable request.
